# Non-Canonical Functions of the ARF Tumor Suppressor in Development and Tumorigenesis

**DOI:** 10.3390/biom11010086

**Published:** 2021-01-12

**Authors:** Nefeli Lagopati, Konstantinos Belogiannis, Andriani Angelopoulou, Angelos Papaspyropoulos, Vassilis Gorgoulis

**Affiliations:** 1 Molecular Carcinogenesis Group, Department of Histology and Embryology, School of Medicine, National Kapodistrian University of Athens (NKUA), 115 27 Athens, Greece; nlagopati@med.uoa.gr (N.L.); kbelogiannis@med.uoa.gr (K.B.); andriani.an22@hotmail.com (A.A.); 2 Biomedical Research Foundation, Academy of Athens, 106 79 Athens, Greece; 3 Molecular and Clinical Cancer Sciences, Manchester Cancer Research Centre, Manchester Academic Health Sciences Centre, University of Manchester, Manchester M13 9PR, UK; 4 Center for New Biotechnologies and Precision Medicine, Medical School, National and Kapodistrian University of Athens, 115 27 Athens, Greece

**Keywords:** ARF, tumor suppressor, non-canonical functions, cancer biology, developmental biology, Hippo pathway, Wnt pathway, Notch pathway

## Abstract

P14ARF (ARF; Alternative Reading Frame) is an extensively characterized tumor suppressor which, in response to oncogenic stimuli, mediates cell cycle arrest and apoptosis via p53-dependent and independent routes. ARF has been shown to be frequently lost through CpG island promoter methylation in a wide spectrum of human malignancies, such as colorectal, prostate, breast, and gastric cancers, while point mutations and deletions in the p14ARF locus have been linked with various forms of melanomas and glioblastomas. Although ARF has been mostly studied in the context of tumorigenesis, it has been also implicated in purely developmental processes, such as spermatogenesis, and mammary gland and ocular development, while it has been additionally involved in the regulation of angiogenesis. Moreover, ARF has been found to hold important roles in stem cell self-renewal and differentiation. As is often the case with tumor suppressors, ARF functions as a pleiotropic protein regulating a number of different mechanisms at the crossroad of development and tumorigenesis. Here, we provide an overview of the non-canonical functions of ARF in cancer and developmental biology, by dissecting the crosstalk of ARF signaling with key oncogenic and developmental pathways.

## 1. Introduction

The CDKN2A locus which is found on human chromosome 9p21 encodes two overlapping transcripts that produce two different proteins, p16INK4a and p14ARF (ARF) [1], both of which are established tumor suppressors. P16INK4a and ARF display no sequence identity, and function through distinct pathways. P16INK4a acts through inhibition of the Cyclin D-CDK4/6 complex, which maintains the Rb protein in its active form [2,3]. On the other hand, ARF acts as a sensor of various types of cellular stress, including oncogenic, heat-shock, and oxidative stress [4,5,6], to subsequently trigger either growth arrest or apoptotic mechanisms in a p53-dependent or independent fashion [7]. Interestingly, ARF has been considered to be an intermediate link between the Rb and p53 pathways, as Rb inactivation leads to increased ARF transcription, which in turn activates a p53-dependent checkpoint [5,8,9].

Early studies on the role of ARF in response to DNA damage had originally dismissed its contribution [10,11], mainly on account of the fact that p53 could still become activated in the absence of ARF, which was however elevated in response to oncogenic stimuli. These initial studies considered the DNA damage and oncogenic response as separate processes with different mediators and outcomes. However, it is now known that oncogene activation can trigger ARF signaling, readily contributing to senescence and cell cycle arrest [5,12,13,14,15,16,17,18]. Importantly, a link was established between ARF and the DNA double-strand break sensor Ataxia Telangiectasia Mutated (ATM), demonstrating that, following genotoxic stress in cancer cells, ATM negatively regulates ARF protein levels [15]. Of note, ARF was also shown to play a role in DNA single-strand break repair [19]. 

Regarding its established role in cancer biology, ARF was identified as a second line of defense against cancer following DNA damage response, with a higher threshold of oncogenic signals being potentially required for its activation [12]. ARF also holds a pivotal role in the Nucleotide Excision Repair (NER) pathway, facilitating the repair of UV-induced DNA lesions [20,21]. Moreover, loss of ARF was also shown to co-operate with BRAF mutations, resulting in increased UV-induced DNA damage and melanoma formation in BRAFV600E mice [22]. Although ARF is well-known for its role in stabilizing p53 levels, including those of mutant p53 [8,23], interestingly, increased ARF expression has been reported in some cases of lung cancer [24], cervical cancer [25], lymphomas [26], and cancer cell lines, such as HeLa and H1299, accompanied by p53 inactivation. Of note, in tumors such as thyroid carcinomas where ARF is upregulated, ARF is unusually found delocalized in the cytoplasm [27]. On the other hand, the role of ARF in the cytoplasm regulating cytoskeleton remodeling and cell adhesion processes has also been postulated [28], thus providing further insight into the pleiotropic roles of ARF in cancer.

In most cases of human cancers, both ARF and p16INK4a are lost, rendering it challenging to define their individual contribution to tumor suppression [9]. Alterations of the complete CDKN2A locus are identified in approximately 30% of human tumors, including glioblastoma, pancreatic cancer, adenocarcinoma, and melanoma [9,29]. The identification of chromatin remodeling events in the CDKN2A locus has recently started to delineate the genetic mechanisms governing expression of ARF and p16INK4a. A cis-element located beside the ARF promoter was recently found to down-regulate p16INK4a via long range interaction, thus providing a clear example of transcriptional regulation facilitated by chromatin folding [30].

Apart from the widely characterized canonical functions of ARF as a barrier to tumor progression, ARF was additionally implicated in other fundamental biological processes, including early development and morphogenesis. In this review, we attempt to discuss the non-canonical aspects of ARF biology by additionally highlighting important links between tumor suppression and developmental biology. We also aim to underline the interplay between ARF and key signaling pathways regulating major aspects of both cancer and stem cell biology. 

## 2. The Role of ARF in Differentiation and Morphogenesis

ARF has been involved in numerous processes regulating cellular differentiation and organ development, such as ocular and mammary gland development, spermatogenesis, and angiogenesis. It was shown that Arf knockout results in the development of smaller eyes with no functional lenses in mice, due to incomplete maturation of the primary vitreous into the secondary vitreous [31]. ARF expression is required for hyaloid vascular system (HVS) involution, a critical process in ocular development, during which ARF prevents the accumulation of mural cells covering the blood vessels and maintains vessel stability through platelet-derived growth factor (PDGF) signaling, independently of p53 [32,33]. ARF was additionally shown to repress PDGF receptor synthesis through microRNA induction [9,34]. Of note, the phenotype of Arf -/- mice bears a strong resemblance with a human congenital ocular disorder termed Persistent Hyperplastic Primary Vitreous (PHPV), where patients also exhibit microphtalmia and lens degeneration [6,31].

ARF has been also found to determine the balance between cellular proliferation and apoptosis during murine mammary gland development [35]. Progesterone-dependent regulation of p19ARF (the p14ARF equivalent in mice) throughout pregnancy results in p19ARF induction, whereas loss of p19ARF leads to a delay in the early phase of involution and decrease in apoptosis through p21 downregulation. A second effect of p19ARF loss seems to be the immortalization of mammary epithelial cells [35]. The study by Yi et al. (2004) concluded that although p19ARF may be dispensable for mammary gland differentiation, its functional interplay with progesterone and p21 is critical for the control of the balance of cell proliferation versus apoptosis, possibly in a p53-dependent manner [35].

The process of spermatogenesis, through which spermatogonia lying on the basement membrane of seminiferous tubules are triggered to differentiate into spermatocytes, has been also found to be under ARF control. ARF is upregulated in mitotically active mouse spermatogonia, while Arf -/- male mice display testicular atrophy accompanied by p53-dependent apoptosis (anoikis) in prophase I, a paradox, given the anti-proliferative effects of ARF in somatic cells [32,36]. The functional link between ATM and ARF remains to be elucidated in male germ cell development, as ARF levels are found downregulated in spermatogonia in response to irradiation [15]. A pivotal role for ARF has been also demonstrated in facilitation of the meiotic divisions leading to spermatocyte maturation [36]. Interestingly, Mu et al. (2014) showed that spermatogonial stem cell self-renewal relies on Polycomb-repressive complex 2 (PRC2) which, through its activity in both mitotic and meiotic germ cells, represses soma-specific genes to facilitate spermatogonial differentiation in an ARF-independent mechanism [37]. 

ARF is not expressed in most fetal or young adult mouse tissues, however it is detected in the fetal yolk sac, a tissue derived from the extraembryonic endoderm (ExEn) [38]. ARF inactivation delays differentiation of the ExEn lineage in embryoid bodies originating either from embryonic stem cells or induced pluripotent stem cells, however, it does not affect the formation of other germ cell lineages [38]. It was shown that ARF functions via p53 to promote differentiation of the ExEn lineage [38]. 

Keratinocyte differentiation is another developmental process implicating ARF signaling. It was found that p63 levels were downregulated while ARF levels increased at the early stages of human keratinocyte differentiation, potentially due to ARF-mediated p63 SUMOylation and subsequent inactivation [39]. Of note, serious skin and skeleton phenotypic defects accompanying the p63-null mouse are partially rectified upon ARF silencing, suggesting that in the absence of p63, aberrant upregulation of ARF may be incompatible with skin development [40].

In addition to its role in cell maturation and organ development, ARF was shown to hold an important role in the control of angiogenesis, a process concerning both normal and pathological conditions. Kawagishi et al. (2010) showed that ARF suppresses Vascular Endothelial Growth Factor A (VEGFA) expression through the p53 pathway in mouse cell lines [41]. Subsequent studies performed in cultured and xenotransplanted human cell lines lacking functional p53 expression showed that following ATM inhibition, an inverse relationship was established between ARF and VEGF expression [6]. Those studies implied that ARF stabilization through ATM inhibition in a p53-deficient background may comprise a potential anti-angiogenic approach against cancer. In line with those observations, ARF was shown to halt angiosarcoma development, potentially in a p53-dependent fashion [42]. Moreover, an increased number of CD31-positive cells in tumors derived from Arf -/- mice strongly suggested new blood vessel formation, while endothelial cells exposed to paracrine signaling from Arf -/- macrophages displayed increased mobility, indicating that ARF deficiency promotes both macrophage infiltration and angiogenesis [43].

## 3. ARF in Stem Cell Biology

CDKN2A expression increases with age, followed by a decline in the tissue regenerative potential. It has been suggested that during the transition from stemness to differentiation, the CDKN2A locus is remodeled to become responsive to stress and mitogenic signals emerging in the differentiation process [9]. The regenerative potential of tissues depends on the balance between stem cell quiescence and self-renewal, both of which are critical processes implicated in tissue homeostasis. Hence, stem cell exhaustion has been considered to be a major hallmark of aging [44,45]. Aging is characterized by accumulation of chronic stress-induced cellular damage, accompanied by higher incidence of tumorigenesis. The ARF/p53 pathway, which is dormant in several tissues throughout development and postnatal life, is progressively activated from adulthood to old age in a wide spectrum of tissues and species [44]. By introducing regulatory and coding sequences of human ARF into the zebrafish genome, it was shown that ARF increases during epimorphic fin regeneration after amputation, contributing to inhibition of the regeneration process [46]. However, inhibition of ARF alone was insufficient to allow regeneration [47]. 

A major mechanism through which ARF contributes to stem cell regulation is through p53 stabilization. While reduced p53 activity is linked to increased stem cell self-renewal, p53 hyperactivation in mouse models has resulted in limited regeneration potential attributed to premature exhaustion of stem cell niches [44]. Thus, depletion of hematopoietic stem cells (HSCs) accompanied by impaired hematopoiesis, disruption of mammary gland morphogenesis, reduction of the neural stem cell pools, and disrupted olfactory functions have been reported in p53 mutant mouse models with higher p53 activity than wild-type counterparts [48,49,50]. In line with those phenotypes, p53 induction in the mouse epidermis through Mdm2 ablation has resulted in compromised stem cell activity and premature skin aging [51]. In contrast, mice with an extra copy of Ink4a/Arf/p53 exhibit extended lifespan and delayed aging, linked to extended preservation of stem cell populations [44,52]. Hence, it has been proposed that moderate and regulated activation of the ARF/p53 pathway during aging yields slower proliferation capacities, likely contributing to stem cell quiescence and ameliorating stem cell aging, by simultaneously preventing the exhaustion of stem cell populations. In support of this notion, p53 or p21 loss in mouse models has resulted in exit from quiescence and long-term depletion of stem cell reservoirs at advanced ages [53,54,55]. 

The polycomb group gene BMI1, which is required for adult stem cell maintenance in many organs [56,57], was found to regulate cell proliferation and senescence through the CDKN2A locus [58]. Jacobs et al. (1999) demonstrated that in Bmi1-deficient mouse embryonic fibroblasts and lymphocytes undergoing premature senescence, the expression of both p16INK4a and p19ARF was markedly increased, while Bmi1 overexpression led to fibroblast immortalization and a decrease in p16INK4A and p19ARF levels [58]. Depletion of the Cdkn2a locus dramatically rescued the phenotypes observed in Bmi1-deficient mice, rendering Cdkn2a critical in vivo Bmi1 target [58]. In line with this observation, it was additionally shown that Bmi1 repressed Ink4a/Arf and Hox genes to allow stem cell self-renewal in rodents [59]. A recent study confirmed the previously reported links between Bmi1 and the Cdkn2a locus, as it demonstrated that the diminished self-renewal capacity of Bmi1-deficient innate-like B lymphocytes was rescued by additional deletion of the Cdkn2a locus [60].

## 4. ARF Crosstalk with Major Signaling Pathways

Apart from its canonical functions as a tumor suppressor, ARF and the ARF/p53 pathway were also found to be directly or indirectly involved in the regulation of other key signaling pathways, which hold important roles in development and tumorigenesis. Those non-canonical functions of ARF signaling were identified through the various types of interactions between ARF and other pathway components or effectors.

### 4.1. The Hippo Pathway

The Hippo pathway is an evolutionarily conserved signaling pathway which actively controls organ growth and development, tissue regeneration, and epithelial homeostasis. Several of those functions are elicited through the terminal effectors YAP and TAZ, which direct gene expression through transcription factor binding [61]. Deregulated Hippo pathway is linked with various diseases, including tumorigenesis, rendering its components promising therapeutic targets. The tumor suppressor RASSF1A, which is epigenetically silenced in the vast majority of sporadic human malignancies, is an upstream regulator of the Hippo pathway, driving the formation of pro-apoptotic YAP–p73 complexes at the expense of pro-proliferative YAP–TEAD complexes [62,63,64]. RASSF1A has been recently found to additionally act as a “molecular switch” required for the transition from pluripotency to differentiation in the pre-implantation mouse embryo, mouse embryonic stem cells, and induced pluripotent stem cells (iPSC) [65]. 

ARF has been previously shown to stabilize the epithelial-to-mesenchymal transition (EMT) marker SLUG in prostate cancer [66], while it also regulates the tumor microenvironment through matrix metalloproteinase-7 (MMP7) nuclear translocation [67]. A potential crosstalk between the Hippo pathway and ARF was discovered when ARF and YAP were found co-elevated in the Pten/p53-null mouse [68]. Subsequent work demonstrated that ARF may deregulate both Hippo and Wnt pathways in human prostate cancer cells [69]. Mechanistically, ARF was shown to deregulate the Hippo pathway by stabilizing cytoplasmic YAP and preventing its nuclear accumulation, potentially through ARF-mediated YAP SUMOylation. Tea-derived carbon nanodots were shown to interact with nuclear ARF to stabilize it, and consequently contribute to YAP cytoplasmic retention, thus providing a potential avenue for targeting the pro-proliferative effects of nuclear YAP complexes [69].

The Forkhead Box (Fox) m1b (FOXM1) transcription factor, which is required for hepatocellular carcinoma (HCC) development, comprises an inhibitory ARF target [70]. Kalinichenko et al. (2004) demonstrated that a p19ARF peptide containing nine D-Arg was sufficient for inhibition of both Foxm1 transcription and Foxm1-dependent growth of osteosarcoma cells, rendering the (D-Arg)9-p19ARF 26–44 peptide a potential FOXM1 inhibitor in tumorigenesis [70]. Interestingly, Hippo pathway deregulation in cancer has been shown to drive FOXM1 activation through YAP–TEAD-dependent FOXM1 transcription, while FOXM1 additionally interacts directly with TEAD on target gene promoters, contributing to cell proliferation and tumor development [71]. Importantly, pharmacological FOXM1 inhibition limits tumor size in vivo, implying that FOXM1 can potentially serve as a therapeutic target in sarcomas [71].

Another potential link between the Hippo pathway and ARF signaling may derive from the fact that the RASSF1A scaffold protein has been shown to inhibit MDM2, similarly to ARF [72]. RASSF1A promotes MDM2 self-ubiquitination and p53 stabilization by disrupting the MDM2–DAXX–HAUSP complex in the nucleus [72]. Although the RASSF1A binding region on MDM2 is distinct from the ARF binding region [73], the E1A-regulated transcription factor E4F1 can physically interact with both ARF and RASSF1A [74,75], suggesting a potential involvement of RASSF1A on the ARF-MDM2 regulation overall. Besides its involvement in p53 stabilization, RASSF1A is also implicated in p53-mediated checkpoint activation in response to DNA damage [72]. Interestingly, RASSF1A was found to be directly activated by ATM upon DNA damage, thus prescribing the Hippo pathway with a pro-apoptotic role via p73 [76,77], whereas ATM was shown to inhibit ARF [15]. Given that RASSF1A is actively involved in YAP–p73-mediated apoptosis, ARF suppression by ATM may be a mechanism employed by cells to prevent ARF-mediated YAP inactivation, a meaningful scenario at least in the case of p53-deficient tumors.

### 4.2. The Wnt Pathway

The Wnt signal transduction cascade is a master regulator of biological processes throughout the development and adult life of all animals. Aberrant Wnt signaling underlies a wide range of pathological conditions in humans, including cancer [78]. The Wnt signaling cascade, which exerts its functions via β-catenin-dependent gene regulation, is an important player in maintaining stem cell self-renewal [78]. Mutations in key components of the Wnt cascade are responsible for the development of various types of cancer, such as melanomas, colorectal, and liver cancers [78,79]. Due to its major involvement in the regulation of stemness, Wnt pathway activation plays a critical role in patient-derived organoid formation, as it has been shown for several tissues such as colon, lung, and skin [80,81,82,83]. Inactivation of the Hippo pathway scaffold RASSF1A was found to be required for Wnt and Hippo pathway crosstalk resulting in maintenance of pluripotency, while RASSF1A uncouples Wnt from Hippo signaling to enable stem cell and embryonic differentiation towards all cell lineages, through p73 [65]. 

**Figure 1:**
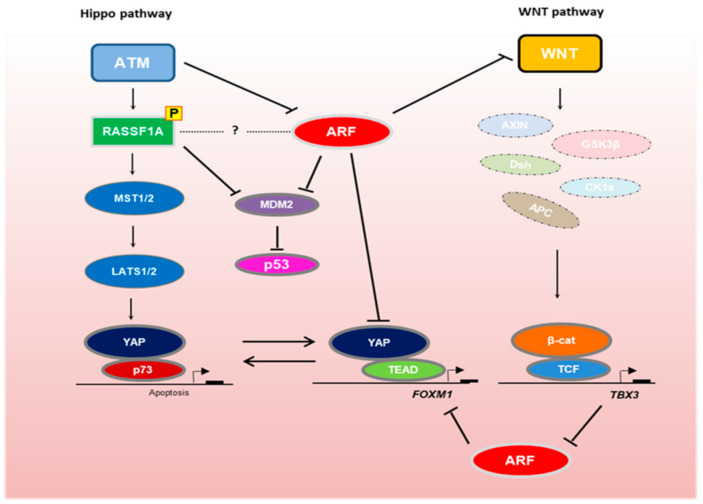
Interplay between the Hippo, Wnt and ARF/p53 signaling pathways in development and tumorigenesis. The Hippo pathway scaffold RASSF1A is activated by ATM-mediated phosphorylation [76,77]. Although ATM inhibits ARF [15], both RASSF1A and ARF prevent MDM2-mediated p53 degradation. RASSF1A activation results in the formation of stable YAP–p73 transcriptional complexes, which drive stem cell differentiation and apoptosis [63,65]. In the absence of RASSF1A, YAP–TEAD complexes drive cellular proliferation via transcription of genes, such as *FOXM1*. FOXM1 is inhibited by ARF [70]. ARF also suppresses Wnt signaling [89,90], which upon activation destabilizes the β-catenin destruction complex, resulting in β-catenin nuclear translocation and TCF/LEF-mediated transcription of oncogenes, such as *TBX3*. TBX3 inhibits ARF to promote oncogenic proliferation [92].

DNA promoter methylation of the p14ARF, RASSF1A, and APC1A genes is considered to be an independent prognostic factor in colorectal cancer patients [84]. Patients with a methylated p14ARF promoter display a significantly worse prognosis, and concurrent methylation of one or more genes from the above set has been linked with poor prognosis independent of tumor stage and differentiation status, suggesting that the pathways involved may synergize towards oncogenic progression and stem cell regulation [84,85] (Figure 1).

Interestingly, the Wnt pathway was found to restrict embryonic stem cell self-renewal via activation of the CDKN2A locus [86]. The Wnt effector Tcf1 is recruited to the Cdkn2a locus of mouse embryonic stem cells (mESC), where it triggers transcription of both p16INK4a and p19ARF, which act as negative regulators of the cell cycle, thereby reducing proliferation rates without affecting the pluripotent status of the cells [86]. The anti-proliferative effect of the Wnt pathway in mESC contrasts with its documented mitogenic effects in somatic cells. 

Damalas et al. (2001) showed that constitutively active β-catenin is capable of inducing an ARF/p53-dependent growth arrest and senescence-like phenotype in mouse embryonic fibroblasts; however, in the absence of either ARF or p53, β-catenin cooperates with Ras to elicit oncogenic transformation [87]. Despite demonstrating β-catenin-mediated activation of ARF and stabilization of p53, the study by Damalas et al. did not show subsequent induction of apoptosis. Other studies suggested that β-catenin likely activates survival pathways to repress the pro-apoptotic effects of the ARF/p53 pathway [16,88].

To identify downstream targets of ARF signaling, gene expression profiling was implemented in human melanoma cell lines with either wild-type or mutant ARF protein [89]. Pathway analysis of the differentially expressed genes between wild-type and mutant ARF cell lines demonstrated that Wnt pathway components may act downstream of ARF signaling, contributing to tumor development when ARF activity is compromised [89]. In line with this, an inverse relationship between ARF and Sprouty RTK Signaling Antagonist 4 (SPRY4), an evolutionarily conserved downstream target of Wnt signaling was observed, as SPRY4 levels increased in the absence of ARF [89,90]. Moreover, TBX3, which was also identified as a downstream target of the Wnt pathway and a pivotal mediator of β-catenin-dependent proliferation and survival in liver tumorigenesis [91], was also found to be upregulated in breast cancer where it represses ARF expression by interacting with histone deacetylases [92]. Of note, the use of HDAC inhibitors was able to rescue TBX3-mediated ARF repression in a dose-dependent fashion, thus providing a potential therapeutic window to disrupt Wnt-mediated ARF suppression [92].

A clear involvement of p19ARF, and not p16INK4a, in tumor escape from growth constraints such as chemotherapy, was demonstrated in a Wnt1-dependent mouse breast cancer model [93]. In order to identify genetic determinants directing tumor escape, a transgenic mouse model was employed, where targeted chemotherapy was simulated by blocking doxycycline-dependent Wnt1 transgene expression. Tumor escape was observed via reactivation of the Wnt pathway [93]. However, Cdkn2a loss, affecting both p16INK4a and p19ARF expression, resulted in tumor relapses with EMT features, without reactivation of the Wnt pathway. Interestingly, p19ARF loss alone enabled rapid EMT-driven tumor escape, while p16INK4a deficiency failed to accelerate relapse, indicating that deregulated ARF/p53 signaling may foster breast cancer relapse regardless of Wnt pathway activation [93]. 

Cdkn2ab knockout mice, deficient for three reading frames (p15INK4b, p19ARF, and p16INK4a), developed a wider spectrum of tumors than Cdkn2a -/- mice, with a higher proportion of skin carcinomas [94]. It was recently shown that the Wnt7b 129P2 allele was sufficient for promotion of oncogenic transformation of Cdkn2ab -/- cell lines via CDK6 activity, an identified Wnt target [95,96]. The recent study by Krimpenfort et al. (2019) suggested that loss of p15INK4b activity, which plays a fundamental role in the hair follicle cell cycle by keeping Wnt-mediated CDK6 activation in check, potentially leads to aberrant progenitor cell migration and proliferation. As p16INK4a and p19ARF present a barrier to cells that respond to these oncogenic signals, concomitant loss of p16INK4a and p19ARF may be a predisposition to skin tumorigenesis [95].

### 4.3. The Notch Pathway

The highly conserved Notch signaling pathway operates in various contexts across which the outcomes can widely differ [97,98]. Notch is a cell surface receptor, formed by intracellular proteolytic cleavage of a single polypeptide chain, whose two deriving subunits undergo dimerization and transport to the plasma membrane [97]. Upon binding of Notch ligands (members of the Delta/Jagged family), the Notch receptor is extracellularly cleaved by an ADAM protease, while intracellular cleavage by γ-secretase results in the release of the Notch Intracellular Domain (NICD) into the cytoplasm, where it enters the nucleus to regulate transcription of target genes [97]. Notch pathway activation can lead to different outcomes depending on the cell type and developmental stage, as it was found to promote differentiation, stem cell maintenance, or tumorigenesis, depending on the set of target genes deployed in each case [99]. Extrinsic signals may, therefore, be important for navigating Notch activity. 

Two of the hallmarks of T-cell acute lymphoblastic leukemia (T-ALL) are aberrant Notch signaling and loss of the CDKN2A locus [100,101]. Interestingly, it has been shown that the intracellular domain of Notch1 results in Arf activation in T-cells, serving as a tumor suppressive mechanism [102]. Arf expression is, however, accompanied by strong selective pressure for deletion of the Cdkn2a locus in T-ALL, thereby leading to the survival of clones bypassing tumor suppression [102]. Subsequent research demonstrated that the Cdkn2a locus is epigenetically silenced in cultured T-ALL initiating cells [103]. However, in T-cell precursors targeted by aberrant Notch1 signaling for transformation, the epigenetic silencing of the Cdkn2a locus was reversed. This enables Arf expression, and again, provides selective pressure for the survival of clones with Cdkn2a loss [103]. Thus, the epigenetic status of Cdkn2a regulates the developmental stages of T-cell maturation where aberrant Notch1 function and Cdkn2a inactivation promote T-ALL [103].

The LIM-only transcription factor (LMO2) gene is subjected to chromosomal translocations in T-cell tumors, leading to abnormally increased LMO2 expression in approximately 9% of all T-ALL cases [100,104,105]. In murine T-cell malignancies, Arf inactivation promotes thymocyte self-renewal and sets the ground for the emergence of aggressive tumors [106]. The Arf promoter is not directly occupied by either Lmo2 or Notch, implying that Notch activation may be a subsequent event in T-cell oncogenesis, and Lmo2 induction functions together with Arf loss to facilitate primitive thymocyte self-renewal [106]. Importantly, aberrant Notch activation and Arf silencing independently cooperate with Lmo2 upregulation to induce T-ALL [106]. Along the same lines, Lmo2 induction promotes the growth of astrocytes isolated from Cdkn2a -/- mice and acquisition of glioma stem cell phenotypes [107]. Intriguingly, and in keeping with the role of ARF in suppressing angiogenesis [6], Lmo2 is capable of inducing angiogenesis in a Cdkn2a -/- background [107].

**Figure 2:**
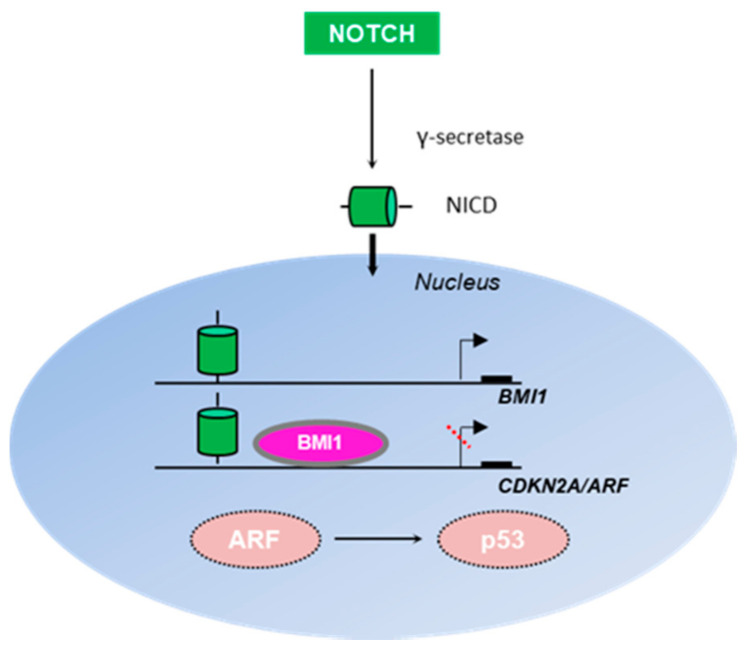
Interplay between the Notch and ARF/p53 signaling pathways. Notch is a transmembrane receptor, which upon cleavage by γ-secretase produces an intracellular domain (NICD) capable of translocating to the nucleus and directly regulating gene transcription. BMI1 is activated by Notch to regulate cellular proliferation and stem cell self-renewal, especially in neural tissue [108]. Both Notch and Bmi1 directly occupy the Arf promoter to repress Arf expression [108], thus perturbing Arf-mediated p53 stabilization.

A critical functional link between the Notch and ARF signaling pathways is the Polycomb group gene BMI1, which actively promotes cellular proliferation and stem cell self-renewal by repressing the CDKN2A locus [58] (Figure 2). BMI1 has been found to be a downstream Notch target, and its loss in mouse intestinal stem cells (ISC) is accompanied by reduced proliferation of the ISC compartment, p16INK4a/p19ARF accumulation, and differentiation towards the goblet lineage mimicking Notch loss-of-function phenotypes [108]. Bruggeman et al. (2005) attempted to explore whether p16INK4a or p19ARF was more critical for eliciting Bmi1 activity, and demonstrated that although p19ARF comprises a general Bmi1 target, p16INK4a derepression plays a role only in a subset of cell types, such as neural stem cells, for the manifestation of Bmi1 -/- phenotypes [109]. The results of the study implied differential cell-type specific functions of p16INK4a versus p19ARF in Bmi-1-mediated cell cycle control [109]. Of note, BMI1 was also shown to promote tumorigenesis independently of ARF in human liver cancer [110]. The tumorigenic activity of BMI1 also appeared to be independent of ARF signaling in human ovarian cancer cells, where genetic or pharmacological BMI1 inhibition impaired clonal growth without affecting expression of the CDKN2A locus or ARF protein stability [111]. The proposed mechanism involved induction of autophagy through ATP depletion, as targeting of BMI1 triggered the PINK1-PARK2-dependent mitochondrial pathway, thereby leading to necroptosis [111].

Notch was additionally reported to suppress p53 in murine T-cell lymphomagenesis through repression of Arf expression, whereas p53 was activated upon Notch signaling perturbation by γ-secretase inhibitors [112]. A subsequent study demonstrated that Notch represses Arf through direct Arf promoter occupancy, where the Polycomb Repressive Complex 2 (PRC2) was recruited in an LSD1-dependent fashion, allowing histone modifications to ultimately terminate Arf transcription (Figure 2) [113]. In contrast, Notch stimulation has been shown to activate p53 in muscle stem cells to promote muscle regeneration via Hey transcription factors [114]. However, retardation of Notch signaling was observed in aged animals, leading to p53 suppression independent of either p16INK4a or p19ARF, thus culminating in a decline in the regenerative potential of muscle cells [114]. 

## 5. Discussion

ARF is a tumor suppressor protein with an established role in important aspects of tumorigenesis, such as the DNA damage response. Apart from its well-known function as a p53 stabilizer, ARF is also implicated in p53-independent mechanisms to promote cell growth arrest [9]. Intriguingly, ARF signaling was also found upregulated in certain tumor types, such as in Burkitt’s lymphoma or the majority of p53 mutant tumors [9,26,115]. To conclusively determine the effects of ARF signaling, ARF still needs to be investigated separately from p16, as the two transcripts of the CDKN2A locus appear to have differing roles in processes such as neural stem cell self-renewal and differentiation [109]. 

In this review, we sought to summarize key findings regarding non-canonical roles of ARF signaling in cancer and developmental biology. In addition, given that development and tumorigenesis are often governed by similar mechanisms, we provide an overview of the crosstalk between ARF alone or the ARF/p53 pathway with major signaling pathways, with pivotal roles in both contexts. The accumulated evidence suggests that ARF is a pleiotropic protein with different regulators and interactors depending on the cell type, developmental stage, and mutation load. By highlighting the interplay between ARF and other pathway components, new links may appear between important signal transduction processes, thus offering the possibility to explore novel therapeutic approaches aimed both at rectifying developmental defects and restricting tumor growth. For example, novel functions identified for the Hippo scaffold RASSF1A in regulating the Wnt pathway [64,65], or the observation that Notch signaling negatively correlates with RASSF1A expression in human tumors such as in the lung and breast [116], in conjunction with RASSF1A- and ARF-mediated p53 regulation, provide a potentially meaningful link between RASSF1A–ARF that deserves to be explored. Along those lines, the Notch transcriptional target BMI1 is a major regulator of ARF expression in development [109], known to hold a neuroprotective role against degenerative disorders such as Alzheimer’s disease [117,118,119]. Hence, the Notch–BMI1–ARF axis constitutes an important clinical target in this field that could be potentially therapeutically exploited.
